# Engaging in Health Behaviors to Lower Risk for Breast Cancer Recurrence

**DOI:** 10.1371/journal.pone.0053607

**Published:** 2013-01-11

**Authors:** Suzanne C. O'Neill, Jessica T. DeFrank, Patti Vegella, Alice R. Richman, Leonard R. Henry, Lisa A. Carey, Noel T. Brewer

**Affiliations:** 1 Lombardi Comprehensive Cancer Center, Georgetown University, Washington, DC, United States of America; 2 Department of Health Behavior, Gillings School of Global Public Health, University of North Carolina, Chapel Hill, North Carolina, United States of America; 3 East Carolina University, College of Health and Human Performance, Department of Health Education and Promotion, Greenville, North Carolina, United States of America; 4 Indiana University Health, Goshen Center for Cancer Care, Goshen, Indiana, United States of America; 5 UNC Lineberger Comprehensive Cancer Center, Chapel Hill, North Carolina, United States of America; Health Canada, Canada

## Abstract

**Purpose:**

While post-treatment breast cancer survivors face up to twice the cancer risk of the general population, modifiable health behaviors may somewhat reduce this risk. We sought to better understand health behaviors that early stage breast cancer survivors engage in to reduce recurrence risk.

**Methods:**

Data came from a cross-sectional multi-site survey of 186 early-stage breast cancer survivors who received genomic testing for breast cancer recurrence risk (Onco*type* DX) during their clinical care. Study outcomes were meeting health behavior recommendations (daily fruit and vegetable intake, regular physical activity, and having a healthy body mass index (BMI)).

**Results:**

Approximately three-quarters of survivors we surveyed believed the 3 behaviors might reduce their cancer risk but many did not engage in these behaviors for this purpose: 62% for BMI, 36% for fruit and vegetable consumption, and 37% for physical activity. Survivors with higher recurrence risk, as indicated by their genomic test results, were no more likely to meet any of the three health behavior recommendations. Adherence to health behavior recommendations was higher for women who were white, college-educated, and had higher incomes.

**Conclusions:**

Many nonadherent breast cancer survivors wish to use these behavioral strategies to reduce their risk for recurrence, suggesting an important opportunity for intervention. Improving BMI, which has the largest association with cancer risk, is an especially promising target.

## Introduction

The numbers of women in the US [1] and worldwide [2] surviving breast cancer has continued to grow with advances in screening and treatment [3]. These survivors face elevated health risks, including cancer recurrence and second primary cancers [4], [5]. The primary tools for reducing recurrence risk are medical interventions, including chemotherapy, radiation and endocrine treatment [6], [7].

Modifiable health behaviors may offer additional reductions in the risk of recurrence and comorbid conditions among cancer survivors, as well as counter the negative impacts of treatment [8]–[13]. Although level I evidence regarding efficacy of intervention is scant, considerable observational and some clinical trial data suggest an impact of health behavior modification in risk of relapse, including maintaining a healthy BMI [14], [15], regular physical activity [16], and a low fat diet [17], [18]. Finally, though evidence for cancer risk reduction from eating fruits and vegetables suggests a very small benefit or none at all [19], [20], these behaviors may support overall health and maintenance of a healthy weight [21], [22]. Guidelines from the American Cancer Society and other national organizations recommend that women with a history of breast cancer eat fruits and vegetables regularly, be physically active and maintain a healthy body weight [23], [24].

Despite this, some breast cancer survivors have poor adherence to these health behavior recommendations. Breast cancer survivors experience obesity at the same high rate as the general U.S. population [25]. Further, in a recent U.S. study, only a little more than a third (37%) of breast cancer survivors were adherent to physical activity recommendations, while only 18% were adherent to recommendations to consume 5 servings of fresh fruits and vegetables daily [26]. Adherence to recommendations is a global issue for survivors, as recent studies also suggest surprisingly low adherence to adjuvant endocrine therapy in breast cancer patients [27], [28]. To better understand how to address low adherence, it is important to understand whether awareness of these behavioral recommendations is low, survivors are disinterested, or if there are other challenges.

Information on recurrence risk may increase women's motivation to follow health behavior recommendations. Women newly diagnosed with breast cancer receive increasingly sophisticated risk information. One example of this includes results of Onco*type* DX, a test that predicts 10-year risk of recurrence among women with early stage breast cancer by examining the activity of a group of 21 genes within a woman's tumor [29], [30]. Breast cancer recurrence risk as determined by this genomic test, as well as a woman's beliefs about risk of recurrence, may play an important role in motivating risk-reducing behaviors. Other potential correlates of adherence include cognitive [31], emotional [32], [33], and motivational constructs [34].

Our study sought to characterize early stage breast cancer survivors' awareness of the potential cancer risk reduction offered by three health behaviors (maintaining a healthy body weight, eating fresh fruits and vegetables regularly, and being physically active). A second goal was to understand whether this awareness was associated with adoption of the behaviors (i.e., adherence to behavioral recommendations). Based on previous research, we expected adherence to these behaviors would be low, but no published research we are aware of has documented whether awareness of the guidelines is a central problem. Last, we also sought to assess variables associated with engaging in these recommended health behaviors. We predicted greater adherence by women who had higher recurrence risk according to their Onco*type* DX test results, who perceived higher recurrence risk and who had more breast cancer worry.

## Materials and Methods

### Participants and Recruitment

Participants were women who were previously treated for early-stage breast cancer at one of three study sites and whose records indicated they received Onco*type* DX testing between the time the test was first available in June 2004 through March 2009. Onco*type* DX testing, which analyzes breast tumor tissue removed during surgery, estimates the 10-year risk for distant breast cancer recurrence assuming the woman receives adjuvant hormone therapy. Women eligible for Onco*type* DX had stages I or II, hormone-receptor positive breast cancer. All but one participant had node-negative disease; we included this woman because the test has proven clinically useful for node-positive breast cancers of the type that she had. Exclusion criteria included being male, not being English-speaking, or under age 18. Study sites included: University of North Carolina (UNC) Breast Center in Chapel Hill, North Carolina; National Naval Medical Center (NNMC) in Bethesda, Maryland; and Georgetown University (GU) in Washington, DC. Participation rates were 75% (78/104), 74% (59/80) and 63% (50/80) for these three sites respectively. We excluded a participant who had an outlier of 194 months since diagnosis, resulting in a combined sample of 186 participants.

Researchers mailed study questionnaires to eligible women between December 2008 and May 2010. The Institutional Review Boards of the University of North Carolina, National Naval Medical Center, and Georgetown University approved the study protocol and materials.

### Measures

#### Adherence to Health Behavior Recommendations

We calculated survivors' body mass indexes using their self-reported height and weight and categorized them as adherent (BMI 18.5–24.9) or non-adherent (25.0 and over or under 18.5). The survey measured adherence to recommendations to consume 5 servings of fruits and vegetables daily: “How many servings of fruits do you usually eat or drink each day? Think of a serving as being about 1 medium piece, or ½ cup of fruit, or ¾ cup of fruit juice,” and “How many servings of vegetables do you usually eat or drink each day? Think of a serving as being about 1 cup of raw leafy vegetables, ½ cup of other cooked or raw vegetables, or ¾ cup vegetable juice.” We categorized respondents who reported consuming 5 or more daily servings across the two measures as adherent. We adapted these items and the physical activity items from The Health Information National Trends Survey (HINTS) [35]. The survey assessed how many days in a typical week survivors engaged in any physical activity of at least moderate intensity, giving the examples of brisk walking, bicycling at a regular pace, swimming at a regular pace, or heavy gardening as well as the duration (in minutes) of this physical activity. We calculated the total minutes spent engaging in at least moderate physical activity in a typical week. We categorized survivors as adherent (150 or more minutes/week) or non-adherent (fewer than150 minutes/week).

#### Cancer Risk-Reduction

We measures cancer risk-reduction awareness by asking survivors to indicate how each of the following behaviors affect chances of getting cancer: 1) having a healthy body weight, 2) eating 5 or more fruits and vegetables each day, and 3) engaging in at least 30 minutes of moderate to vigorous physical activity on 5 or more days each week. Response options were “decreases chances,” “makes no difference,” “increases chances,” and “don't know.” We categorized women who indicated that these behaviors decreased chances as “aware” with the latter responses constituting the “unaware” group.

We assessed engagement in cancer risk-reduction behaviors (BMI, fruit and vegetable consumption, physical activity) by asking survivors for each of the three health behaviors, whether they engaged in any of those behaviors to specifically reduce their chances of getting cancer or a recurrence. Response options were “Yes,” “No,” and “No, but I want to.”

We assess stage of change as has been done in previous research on health behavior in other populations [36]. We categorized as “Unaware” answers to the cancer risk-reduction awareness question of “makes no difference,” “don't know,” or “increases chances.” We classified responses of “decrease chances” as “Aware.” We further categorized women in this group as 1) “Aware, not engaging” if they were not engaging in the behavior, 2) “Aware, engaging, but not to reduce cancer risk,” if they were engaging in the behavior but answered “No” or “No, but I want to” to the cancer risk-reduction behavior question, or 3) “Aware, engaging to reduce cancer risk” if survivors were engaging and doing so specifically for cancer risk reduction.

#### Breast Cancer Recurrence Risk

The survey assessed survivors' recollection of the likelihood of their cancer coming back based on results of their Onco*type* DX test as “low” “intermediate” or “high” risk. We collapsed the intermediate and high risk groups for analyses due to small numbers who reported their risk was high. We previously have found these self-reports match clinical records with a high degree of accuracy [37], [38]. We also asked survivors to report their perceived risk of recurrence on a 0–100% scale [39].

#### Breast Cancer Worry

The survey assessed how often women worried during the past few weeks about their breast cancer recurring, measured on a 4-point response scale ranging from “not at all” to “all of the time” [40].

### Data Analyses

We generated descriptive statistics to characterize the sample and the proportions of survivors in each stage of behavior change for each of the three health behaviors. We conducted bivariate analyses to identify demographic, medical and psychosocial variables associated with meeting each of the three health behavior outcomes (yes/no) using logistic regression. We then conducted separate multivariate logistic regression analyses for meeting each BMI, diet, and physical activity outcome, including variables identified as statistically significant at *p*<.10 in bivariate analyses. We analyzed data with two-tailed statistical tests with a critical alpha of .05 in SAS.

## Results

### Study Sample

On average, women were 57 years old (range 30–84) and were 23 months since diagnosis (range 4–68). Most were Caucasian (80%), college educated (61%), and had a household income of at least $60,000 (69%) (Table 1). Most (72%) reported a low Onco*type* score, while fewer reported intermediate (24%) or high (4%) scores. Women's mean perceived recurrence risk was 16% and their overall breast cancer worry was low (M = 1.8).

**Table 1:** Participant Characteristics (*n* = 186).

	% (*n*) M (SD)
**Diagnosis and Treatment**	
Self-reported Oncotype Recurrence Risk	
Low (<12%)	72 (113)
Intermediate (12%–21%)	24 (37)
High (>21%)	4 (7)
Time since breast cancer diagnosis (months)^1^	22.62 (15.9)
<13	30 (56)
13–24	36 (66)
≥25	34 (63)
Treatments received	
Radiation	63 (118)
Chemotherapy	30 (55)
Hormone therapy	79 (144)
Chemotherapy and Radiation	17 (32)
Body mass index (kg/m^2^)	26.28 (5.90)
Underweight	2 (3)
Normal weight	49 (89)
Overweight	27 (49)
Obese	23 (42)
**Sociodemographics**	
Age (mean)	56.62 (9.92)
Race/Ethnicity	
White	80 (146)
Black	14 (26)
Other	6 (10)
Education	
Less than college	39 (73)
College graduate	61 (112)
Married or living as married	70 (130)
Health Insurance	97 (177)
Annual household income	
<$60,000	31 (51)
≥$60,000 or more	69 (116)
**Psychosocial**	
Perceived Recurrence Risk	15.73 (18.5)
Breast Cancer Worry^2^	1.80 (0.91)
**Adherence to Health Behaviors**	
BMI	49 (89)
Regular fruit and vegetable intake	47 (87)
Regular physical activity	51 (93)

*Note*: Some variables reflect an *n*<186 due to missing data for that variable;

1 We excluded a participant with an outlier of 194 months from all analyses;

2 Breast cancer worry items had a response scale coded 1–4, with higher scores reflecting greater worry about cancer recurrence.

### Stage of Adoption

Most women were aware that the three health behaviors could influence cancer risk (Table 2). Lack of awareness was similar for having a healthy body weight (17%), fruit and vegetable consumption (23%), and physical activity (21%). However, awareness that health behaviors could influence cancer risk did not translate into high rates of engaging in the behaviors. About 62% of women reported that they did not have a healthy body weight despite their awareness that maintaining a healthy weight could reduce risk. About one-third of women reported that they did not engage in the recommended behaviors for physical activity (37%) or fruit and vegetable consumption (36%), despite their awareness that these behaviors potentially reduce risk. Smaller percentages were aware and engaging, but not specifically to reduce their cancer risk. A relatively small percentage of the sample was aware and specifically reported having a healthy body weight (12%) and engaging in fruit and vegetable consumption (21%) and physical activity (18%) to reduce their cancer risk.

**Table 2:** Stage of Behavior Change.

	% Unaware	Aware that health behavior reduces cancer risk		
Self-Reported Health Behavior		% Aware but not engaging	% Aware and engaging, but not to reduce cancer risk	% Aware and engaging to reduce cancer risk
Have a healthy weight (*n* = 184)	17	62	9	12
Consume 5 or more fresh fruit and vegetable per day (*n* = 185)	23	36	20	21
Engage in moderate-intensity physical activity 150 or more minutes per week (*n* = 182)	21	37	24	18

### Correlates of Adherence

Looking across awareness categories, reported adherence to the three behaviors was 49% for having a healthy body weight, 47% for regular fruit and vegetable consumption, and 51% for physical activity. Only 16% of the participants met recommendations for all three behaviors combined. Contrary to our hypothesis, rates of adherence to the three health behaviors were equivalent across women's self-reported Onco*type* risk category (all *Ps*>0.42) (Table 3). About one-half of women met recommendations for the three health behaviors regardless of whether their Onco*type* DX scores indicated low versus intermediate/high risk.

**Table 3:** Correlates of Meeting Health Behavior Recommendations-Breast cancer information.

	Healthy Body Mass Index		Fruit/Vegetable Consumption		Physical Activity	
	% Adherent	OR (95% CI)	% Adherent	OR (95% CI)	% Adherent	OR (95% CI)
**Breast Cancer information**						
Oncotype recurrence risk						
Low	47	ref	45	Ref	54	Ref
Intermediate/High	47	0.97 (0.48–1.96)	52	1.33 (0.66–2.68)	51	0.89 (0.44–1.80)
Months since br ca dx	–	1.01 (0.99–1.03)	–	0.99 (0.97–1.01)	–	1.01 (0.99–1.03)
Chemotherapy treatment						
No	55	ref	42	Ref	53	Ref
Yes	33	0.41 (0.21–0.79)**	58	1.92 (1.02–3.64)*	46	0.75 (0.40–1.42)
Radiation treatment						
No	51	ref	47	Ref	52	Ref
Yes	47	0.88 (0.48–1.60)	46	0.98 (0.54–1.79)	51	0.95 (0.52–1.73)
Hormone therapy						
No	50	ref	45	Ref	41	Ref
Yes	49	0.96 (0.47–1.96)	47	1.11 (0.54–2.27)	54	1.74 (0.84–3.64)

***Notes.*** Analyses are bivariate. OR = odds ratio. CI – confidence interval. Br ca dx = breast cancer diagnosis. BMI = body mass index.

* *p*<.05,

** *p*<.01,

***p<.0001,

**§** *p*<.10.

In bivariate analyses, women who received chemotherapy as part of their care were less adherent to BMI recommendations compared to women who did not receive chemotherapy but more adherent to recommendations related to fruit and vegetable consumption. White women were more likely to be adherent to BMI recommendations when compared to non-White women (Table 4). Women with a college degree or higher were more likely to be adherent than non-college-educated women across all three behaviors, while women who reported household incomes higher than $60,000 were more likely to be adherent to recommendations for BMI and fruit and vegetable consumption. Awareness of associations between each health behavior and lower cancer risk was only related to adherence for fruit and vegetable consumption. Adherence was unrelated to perceived risk or cancer worry.

**Table 4:** Correlates of Meeting Health Behavior Recommendations-Demographics and Psychosocial variables.

	Healthy Body Mass Index		Fruit/Vegetable Consumption		Physical Activity	
	% Adherent	OR (95% CI)	% Adherent	OR (95% CI)	% Adherent	OR (95% CI)
**Demographics**						
Age						
<55	55	ref	46	Ref	53	Ref
≥55 years	43	0.63 (0.35–1.14)	47	1.04 (0.58–1.87)	50	0.88 (0.49–1.60)
Race						
White	56	Ref	48	Ref	51	Ref
Black or other	22	0.23 (0.10–0.53)**	42	0.78 (0.37–1.62)	51	1.00 (0.48–2.10)
Education						
<College	31	Ref	34	Ref	42	Ref
≥College	59	3.27 (1.74–6.13)**	55	2.38 (1.29–4.38)**	57	1.81 (0.99–3.33)§
Marital status						
Not Married	42	ref	40	ref	47	ref
Married	51	1.46 (0.77–2.76)	50	1.50 (0.79–2.84)	53	1.25 (0.66–2.38)
Annual household income						
<$60,000	29	ref	33	ref	51	ref
≥$60,000	56	3.07 (1.52–6.23)**	55	2.46 (1.24–4.90)*	51	1.01 (0.51–1.99)
BMI						
Normal weight	–	–	52	ref	57	ref
Overweight/obese	–	–	39	0.60 (0.33–1.08)§	45	0.61 (0.34–1.10)
**Psychosocial**						
Perceived breast cancer recurrence risk	–	1.00 (0.98–1.01)	–	0.99 (0.97–1.01)	–	1.00 (0.99–1.02)
Breast cancer worry	–	0.92 (0.67–1.27)	–	0.77 (0.55–1.07)	–	0.92 (0.67–1.27)
Awareness that this behavior reduces cancer risk						
No	52	ref	26	ref	46	ref
Yes	48	0.87 (0.40–1.88)	53	3.20 (1.49–6.85)**	53	1.32 (0.64–2.71)

***Notes.*** Analyses are bivariate. OR = odds ratio. CI – confidence interval. Br ca dx = breast cancer diagnosis. BMI = body mass index.

* *p*<.05,

** *p*<.01,

***p<.0001,

§ *p*<.10.

In the multivariate model for BMI adherence, race (OR = 0.28, 95%CI = 0.11–0.75, *p*<.05), education (OR = 3.06, 95%CI = 0.14–6.73, *p*<.01), and chemotherapy (OR = 0.28, 95%CI = 0.13–0.62, *p*<.01) remained statistically significant. In the multivariate model for fruit and vegetable consumption, education (OR = 2.21, 95%CI = 1.01–4.79, *p*<.05), chemotherapy (OR = 2.50, 95%CI = 1.14–5.47, *p*<.05), and awareness (OR = 3.96, 95%CI = 1.59–9.87, *p*<.01) remained statistically significant. In the multivariate model for physical activity, adherence had no statistically significant correlates.

## Discussion

Most survivors were aware that fruit and vegetable consumption, engaging in physical activity and having a healthy body weight could potentially reduce their cancer risk. However, despite this awareness, over one-half of survivors said they were not maintaining a healthy body weight to reduce their cancer risk, and about one-third said they were not engaging in diet and exercise behaviors to reduce their cancer risk.

We additionally found disparities in adherence by race and level of education. Women with less education were less adherent to recommendations for BMI and fruit and vegetable consumption. Differences by race occurred only for BMI, with African American women being less likely to be adherent to BMI recommendations than White women. A substantial literature shows higher BMI among African American survivors than other survivors in the population [41]–[43]. However, the impact of obesity on recurrence and survival among African Americans is unclear. A recent study of almost 5,000 breast cancer survivors under 65 suggests that obesity is a risk factor for breast-cancer related and all-cause mortality for White but not African American breast cancer survivors [44]. Nonetheless, obesity remains an important concern for all survivors, as it is a risk factor for other breast-cancer related complications, such as lymphedema, which can lower women's quality of life [42], [45]. Differences also were observed for those who had received chemotherapy. Chemotherapy itself alters metabolism, and weight gain during adjuvant chemotherapy is common in breast cancer patients, so a nonvolitional component may play a role in this group. In sum, our findings suggest that intervention efforts among breast cancer survivors that encourage health behaviors should make use of survivors' existing enthusiasm for action rather than promoting awareness. Given that enthusiasm was highest for BMI reduction and evidence suggest the strongest link between this behavior and lower recurrence risk, interventions should prioritize this outcome. Recent intervention literature has focused on multiple pathways to health behavior change and weight management, including both increasing knowledge and awareness [46], [47] as well as incorporating self-regulation skills [48] and skill building [49], [50] that would support action. Given the identified disparities, these interventions should be inclusive of women with varying levels of education and should be culturally sensitive [42].

Contrary to our hypothesis, survivors' who reported intermediate or high recurrence risk, as provided by their genomic test results, were no more likely to meet recommendations for the three health behaviors than other women. Adherence was also unrelated to perceived risk of recurrence or cancer worry. Rates of adherence could have been higher for the higher risk women immediately following diagnosis, but we were unable to capture this with our retrospective design and these outcomes were unrelated to time since diagnosis. We speculate that, for women with intermediate to high risk for cancer recurrence, engaging in health behaviors to reduce their risk is not a priority. Rather, these women may be relying on medical risk-reducing strategies as advised by their doctors, such as receiving chemotherapy and endocrine therapy to reduce recurrence risk. Both treatments have substantially stronger evidence for reducing cancer recurrence risk than health behaviors. However, women may additionally benefit from health behavior change, as these are viewed by clinicians as healthy lifestyle changes that are encouraged for a variety of reasons. In keeping with public health approaches that emphasize at-risk populations, future intervention efforts could focus on survivors at higher risk for cancer recurrence based on their genomic test results or other common pathological markers (such as tumor size or stage) as they are no more likely to engage in these health behaviors. Survivors with high recurrence risk would additionally benefit from improved adherence to health behavior recommendations given the high rate of chemotherapy use in this population, as these health behaviors could ameliorate some sequelae of chemohormonal therapy [51].

Strengths of our study are its multi-site sample of breast cancer survivors for whom the findings are clinically relevant. Women in our study were among the first to receive the Onco*type* DX test and thus positioned us to ask novel questions about the influence of genomic testing on health behaviors. Further, to our knowledge, this is the largest study to date to include self-report data from women who have received this testing. Despite these strengths, we were limited by our cross-sectional, self-report data and that women were, on average, almost two years from diagnosis. We also do not know the degree to which lifestyle counseling was part of the care received by the participants in this study. Finally, though findings for fruit and vegetable consumption's effect on recurrence risk are equivocal, eating these foods regularly substantially reduces cardiovascular risk [21], [22], a topic for future study.

## Conclusions

Our findings corroborate previous research with cancer survivors suggesting that adherence to health behaviors for this population is less than optimal and that there are significant disparities in adherence. While cancer survivors are aware that health behaviors may play some role in reducing cancer recurrence, this awareness often does not translate to action. Interventions should focus on strategies to facilitate actions, such as providing survivors with the appropriate skills and resources to achieve a healthy lifestyle, with a special focus on BMI reduction [52].
